# The Role of Neutrophil CD11b Compared to Neutrophil CD64 as an Early Diagnostic, Monitoring, and Prognostic Sepsis Marker in Neonatal ICUs: Case-Control—Methodological Study

**DOI:** 10.1155/bmri/7206112

**Published:** 2025-03-12

**Authors:** Heba E. Hashem, Wafaa O. Ahmed, Safeya H. Hassan

**Affiliations:** Clinical Pathology Department, Ain Shams University Hospitals, Cairo, Egypt

**Keywords:** biomarkers, diagnostic, inflammatory, neonates, neutrophil CD11b, neutrophil CD64, sepsis

## Abstract

**Background:** Early diagnosis and treatment of neonatal sepsis are crucial to cut off its major medical consequences: lifelong morbidities, neurodevelopmental disabilities, and a high number of neonatal mortalities.

**Aim of the Work:** This study is aimed at determining the diagnostic and prognostic performance of CD11b as a sepsis biomarker for detecting neonatal sepsis at early stages compared to nCD64 and the other conventional sepsis parameters.

**Methods:** Two hundred eleven neonates were enrolled from three Egyptian neonatal ICUs (NICUs), and they were classified into two main groups: the control group (*n* = 101) and the sepsis group (*n* = 110). Enrolled neonates were subjected to full sepsis screening, including complete blood count (CBC), C-reactive protein (CRP), blood cultures, and flow cytometry analysis for both CD64 and CD11b on the neutrophil surface (results represented as a percentage (percent) and mean fluorescent intensity (MFI) units for either biomarker).

**Results:** nCD64% (median = 44.15%) was significantly enhanced in the sepsis group compared to the controls (median = 25%), achieving 90.8% specificity, 92.8% sensitivity, and AUC = 0.894, respectively. CD64 MFI and CD11b MFI could differentiate between sepsis and control groups but with low undesirable diagnostic performance (sensitivity: 72.5% and 59.1%; specificity: 54.4% and 69.4%; AUC: 0.634 and 0.144, respectively). CD11b% could not discriminate between sepsis and control neonates (sensitivity and specificity of 31.8% and 73.6%, respectively) with an AUC of 0.405. hs-CRP had moderate diagnostic performance, achieving sensitivity and specificity of 69% and 78.15%, respectively, and AUC = 0.586. ROC analysis showed that combined hs-CRP and CD64% results had the highest sensitivity and specificity in the current study, being 93.9% and 97.2%, with AUC = 0.938, respectively.

**Conclusion:** CD64%, CD64 MFI, CD11b MFI, and hs-CRP are increased in neonates with sepsis comparable to the controls. CD64% has a superior diagnostic performance comparable to nCD11b and hs-CRP. Combined nCD64 with hs-CRP measurement can provide rapid and accurate diagnostic modality for sepsis diagnosis in correlation with the patient's clinical condition and context with the results of other hematological indices; neutrophil CD64 can be routinely applicable in NICUs for better sepsis management. It is statistically evident that nCD11b is less ideal compared to nCD64 as a diagnostic, prognostic, or monitoring sepsis marker.

## 1. Introduction

Sepsis and septic shock represent distinct but related entities. Infection is generally used as an umbrella term for any suspected or proven infective process, regardless of etiology or severity [1]. Within pediatrics, sepsis has classically been defined as a syndrome of systemic inflammation with resultant clinical or laboratory abnormalities in the presence of proven infection, most commonly a positive blood culture [1].

Neonatal sepsis represents a major cause of neonatal morbidities and mortality worldwide [2]. Early diagnosis is crucial to cut off subsequent neonatal complications. Neonatal septicemia is usually classified into early-onset sepsis (EOS) and late-onset sepsis (LOS), where EOS occurs in the first 72 h of life and is transmitted perinatally from the mother, while LOS occurs in the subsequent days of life, being acquired after delivery from postnatal sources [3].

Many risk factors predispose to neonatal infections; premature rupture of membranes (PROMs) is considered a major risk factor, and neonates are usually colonized with the microflora of the maternal birth canal after ROM, especially if it occurs more than 24 h preceding the time of delivery or is acquired as an ascending infection. Prolonged ROM results in chorioamnionitis, representing the breakdown of the protective maternal barrier against maternal microflora, and usually manifests as EOS [4].

Other routes of infection are bloodstream-borne and direct routes from the surrounding environment during invasive procedures in neonatal ICUs (NICUs), such as hyperalimentation infusions, assisted ventilation, vascular access, and urinary catheter insertion. This is followed by transient bacteremia that may progress to the multiplication of the organisms and invasion of the bloodstream, which usually manifests as LOS [5].

The early symptoms and signs of neonatal sepsis vary and are mostly nonspecific, so it is easy to be misdiagnosed [6]. To date, no single ideal laboratory marker for sepsis diagnosis is available that can allow the clinician to depend upon its result to confirm his diagnosis. Although positive blood cultures can be used as the gold standard for sepsis diagnosis, however, its results have undesirable sensitivity, are time-consuming, do not frequently correlate with the clinical condition of the neonate, and cannot differentiate EOS from LOS [7]. So, the need for a rapid and sensitive test is raised to obtain a reliable indication of neonatal infections and to differentiate EOS from LOS, hence allowing for tailoring the management protocols accordingly.

Recent advances in flow cytometric technology have provided opportunities for detecting cell surface activation markers on specific cell types in a few hours with a minimal blood sample volume. Both neutrophil CD11b (nCD11b) and neutrophil CD64 (nCD64) are present at the top of the list of the studied cell surface sepsis biomarkers [8–12]. They are proteins of *β*-integrin adhesion molecules, and they have a vital role in innate immunity by defending against infection through stimulation of neutrophil migration to the sites of infection, hence clearing the infected site from the invading microorganisms [13].

Both nCD64 and nCD11b appear on the neutrophil surface within a few minutes of bacterial exposure. Due to their reported high sensitivity and specificity, they can be used as early diagnostic biomarkers for neonatal sepsis [7]. Both biomarkers increase markedly within a few minutes after the cells are exposed to bacteria or endotoxins, and this unique advantage justifies the use of either of them as early markers for the detection of bacterial infections [14–16].

Several studies documented that cell surface antigens such as CD11b and CD64 can be used in routine investigations for the early diagnosis of neonatal sepsis. Such usage in combination with acute-phase reactants may enhance diagnostic accuracy [6, 7, 17]. So, our study was aimed at evaluating the diagnostic and monitoring performance of both biomarkers as diagnostic sepsis markers at the early stages of the disease compared to each other and comparable to the conventional sepsis diagnostic laboratory methods and as a secondary objective to evaluate the predictive performance of studied biomarkers for mortality in aim to decide which is the best fitting for the routine clinical application.

## 2. Materials and Methods

### 2.1. Study Design

Two hundred eleven neonates were enrolled in the current study during the period from April 2020 to November 2023; they were selected from neonates who were admitted to the NICU in Children's Hospital, Ain Shams University Hospital, Cairo, Egypt. According to the neonate's clinical and laboratory findings, they were selected based on physician discretion. Ethical approval: The study underwent a thorough evaluation and received permission from the research ethics committee, Faculty of Medicine, Ain Shams University (Reference Number: FMASU R32/2023) and from the concerned institutional research ethics committee. The study is a case-control–methodological study that followed the Code of Ethics of The World Medical Association (Declaration of Helsinki) for studies done on humans. Informed parental consent was obtained.

### 2.2. Inclusion Criteria

Neonates at different gestational ages (GAs), dated as per the New Ballard Score, were enrolled in the study according to the New Ballard Score [18]. For sepsis patient identification and selection, neonates had presumed one or more infection risk factors, including a history of prematurity, low birth weight, PROM more than 24 h, maternal peripartum fever, or maternal urinary tract infection [19], in addition to at least two clinical and two laboratory criteria described in previous literature [6, 8, 20]. These criteria included poor peripheral perfusion, hemodynamic instability, hypothermia, fever, respiratory compromise, intercostal retractions, tachypnea, grunting, or desaturation, poor activity, fits, abdominal distension, feeding intolerance, bleeding, tachycardia, bradycardia, hypoglycemia, aspiration, poor suckling, and lethargy.

Regarding the laboratory criteria for inclusion, it included the following: (i) white blood cell (WBC) count < 5 or > 20 × 10^9^ cells/L, (ii) immature to total neutrophil (I:T) ratio > 0:2, (iii) platelet count < 100 × 10^9^/L, and C-reactive protein (CRP) > 10 mg/L. The diagnosis was verified thereafter by positive blood culture [21].

The controls were subjected to routine clinical examination, and sepsis screening results were negative; they were healthy control neonates selected from the obstetric and gynecological unit. They were sex and weight-matched with the patients.

### 2.3. Exclusion Criteria

Neonates with an inborn error of metabolism or birth asphyxia were excluded from the study.

### 2.4. Patients' Classifications

Selected neonates were divided into the sepsis group (Group 1) (*n* = 110) and the control group (Group 2) (*n* = 101). Group 1 was further classified into two subgroups (Group 1a for EOS neonates (39 neonates) and Group 1b for LOS neonates (71 neonates). Sepsis patients (*n* = 110) were reclassified based on the severity of the disease into two groups: those with severe sepsis/septic shock (*n* = 37), who met the clinical criteria for disease severity, and those with nonsevere sepsis (*n* = 73). The severity of neonatal sepsis was determined by the presence of sepsis plus one of the following: cardiovascular organ dysfunction, acute respiratory distress syndrome, or two or more other organ dysfunctions. Septic shock is the next stage of the sepsis continuum, which occurs when multiple inotropes are used without clinical benefit, followed by neonatal death.

In addition to diagnostic and prognostic sepsis evaluations, enrolled neonates underwent follow-up assessments. On the fifth day following the baseline evaluation, the second clinical and laboratory evaluations were performed. The neonates were divided into two groups based on their clinical condition (Group 1: nonimproved sepsis patients and Group 2: improved sepsis patients).

Additionally, for predictive performance evaluation, the subjected patients were reclassified according to their outcome into the survivors' group and the nonsurvivors' group.

### 2.5. Patient's Evaluation

The selected neonates were subjected to general examination and laboratory sepsis evaluation (as early as sepsis was suspected clinically), including CBC, highly sensitive C-reactive protein (hs-CRP), and blood cultures. Further sepsis evaluations included various cultures like stool, urine, cerebrospinal fluid, and central venous catheter cultures, besides radiological assessments like a chest x-ray and abdominal ultrasonography obtained.

Ethylenediaminetetraacetic acid (EDTA) whole blood specimens were used for CBC analysis (samples were analyzed by the Coulter LH750 analyzer [Coulter Corporation, United States]), and the remaining blood was used for cell surface marker (CD11b and CD64) analysis on the neutrophil population by flow cytometry technology (50 U sample volume/test). CBC samples were analyzed by the Coulter LH750 analyzer (Coulter Corporation, United States).

Serum CRP was measured by the dimension clinical chemistry system. The nephelometric method (hs-CRP technique) was used. A concentration below 6 mg/L was considered a negative result according to laboratory references in our hospital.

Blood culture was performed by using blood culture bottles (BacT/Alert SA BioMerieux Inc., Durham, NC27704), then incubated at 37**°**C until a positive signal was given or up to 7 days to be discharged as a negative result. Subculture was done on blood agar, chocolate agar, and MacConkey's agar, and identification of bacteria using the VITEK 2 (BioMérieux, Marcy l'Etoile, France) automated microbiology system was performed.

For CD64 and CD11b laboratory assessment, EDTA samples were preserved at 4**°**C till analysis (maximum of 48 h), when they were further stained with mAbs and respective isotypic controls. The mAbs (Immunotech, Marseille, France) included anti-CD11b (clone: bear1, isotype: IgG1 mouse; FITC) and anti-CD64 (clone: 22, isotype: IgG1 mouse; PE) were used. All samples were fixed and analyzed by flow cytometry on an Epics XL-MCL (Coulter) with a turnaround time (TAT) of CD markers 72 h and 125 L.E. as cost/test. Neutrophils were screened by their light scatter pattern. Results were expressed as a percentage (percent) and mean fluorescent intensity (MFI) of cells showing expression of the tested adhesion molecules.

### 2.6. Statistical Analysis

Statistical analysis was performed using the SPSS statistical software package (IBM SPSS Statistics V. 24.0). The results of the parametric data were analyzed by using a Student's *t*-test and expressed as mean ± SD, while quantitative nonparametric data were expressed as median and interquartile range using Wilcoxon's rank-sum test. Qualitative data were expressed as numbers and percentages. In addition to the correlation statistics (Spearman's correlation), which were conducted for the possible associations between every two studied variables, the significance threshold was used for statistical tests, as *p* values less than 0.05 were considered statistically significant, and *p* values less than 0.01 were considered highly significant. The diagnostic specificity and sensitivity were tested using receiver operating characteristic (ROC) curve analysis, and the threshold selection for sensitivity/specificity that achieved more than 50% for each parameter was selected. Multiregression analysis was constructed to test the best panel of all the studied laboratory parameters that can be routinely applicable. The normal distribution of variables was evaluated using the Kolmogorov–Smirnov test.

## 3. Results

Enrolled neonates were classified into the sepsis group (Group 1) (*n* = 110) and the control group (Group 2) (*n* = 101). Case and control groups were sex- and weight-matched (*p* values 0.804 and 0.168, respectively). There were significant differences between sepsis patients (both EOS and LOS groups) compared to the controls regarding the GA being lower in the sepsis groups (*p* value 0.03), reflecting the prematurity as a major risk factor.

Additionally, the need for respiratory support, surgical interventions, and the presence of congenital anomalies was much higher among sepsis patients compared to the controls (*p* < 0.001). Duration of hospitalization (DOH) was significantly longer in the case group compared to the controls (*p* < 0.001) as they needed a prolonged hospital stay until medical improvement and management of sepsis complications.  Table 1 summarizes the demographic and laboratory characteristics of the three studied groups.

**Figure 1:**
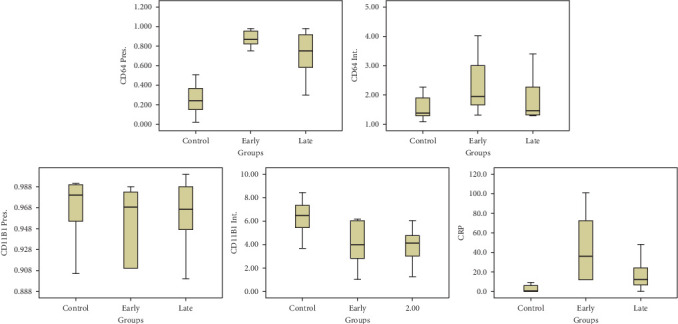
Box plot of CD11b%, CD11b intensity, CD64%, CD64 intensity, and hs-CRP in the three studied groups.

The following figure demonstrates the box plot of CD64%, CD64 MFI, CD11b%, CD11b MFI, and hs-CRP in the three studied groups (Figure 1).

Table 2 demonstrates that CD64% at a cutoff value of 44.15% achieved 92.8% sensitivity and 90.8% specificity, respectively, while CD64 MFI at a cutoff value of 1.43 achieved 72.5% sensitivity and 54.4% specificity. Both nCD11b MFI and nCD11b% documented a lower diagnostic performance than nCD64. The best efficacy achieved in the current study was documented by the combined measurement of hs-CRP and CD64%, reaching 93.9% sensitivity and 97.2% specificity, respectively.

**Figure 2:**
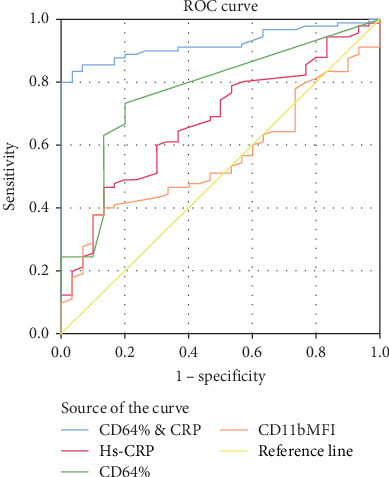
ROC curve analysis of mentioned parameters for discriminating patients with sepsis from those without.

Figure 2 illustrates the ROC curve analysis of the studied parameters for discriminating patients with sepsis from those without and their best combination.

Table 3 demonstrates the third model of multiregression analysis that was constructed to detect the best panel of sepsis markers that can achieve better results for the diagnosis. The previous models included the other potential confounding variables studied to reduce bias, but the result *F* ratio was lower than the presented model. Combined measurement of CD64%, hs-CRP, TLC, and ANC achieved the highest *F*-ratio (24.0) with a highly significant value (*p* value < 0.001) between sepsis and control groups.

CD64 expression showed a significant increase in neonates with positive culture compared to neonates with negative culture results (*p* value = 0.04), while the remaining sepsis parameters remained unchanged between both studied groups. Regarding the comparison between EOS and LOS and the ability to differentiate between both clinical groups, both CD64 and CD11b failed to show a significant difference between both groups, while hs-CRP achieved a significant difference between both groups (*p* value 0.04).

Concerning prognostic sepsis evaluation, sepsis patients were reclassified into the severe sepsis group (*n* = 37) and nonsevere sepsis group (*n* = 73); the comparison was conducted between both groups, and the results are illustrated (Table 4).

Concerning the significant study correlations, hs-CRP was negatively correlated with the platelet count in the LOS group (*p* value 0.002, *r* = −0.598), a significant positive correlation between hs-CRP and DOH can be documented (*p* value 0.04, *r* = 0.399), and both correlations signify the importance of hs-CRP as a prognostic sepsis marker. CD11b% was negatively correlated with DOH in the EOS group (*p* value 0.042, *r* = −0.829), and no significant correlation has existed between either CD64 and CD11b together or between hs-CRP with any of those two markers except for the (HS) correlation between CD64% with CD64 MFI (*p* value < 0.001, *r* = 0.881) and CD11b% and CD11b MFI (*p* value 0.002, *r* = 0.632).

### 3.1. Monitoring Performance of Analyzed Sepsis Biomarkers

After 5 days from the baseline evaluation, 62 of the recruited neonates had a follow-up assessment. Clinically, they were divided into two groups: Group 1 (patients with improved sepsis) (*n* = 42) and Group 2 (patients with nonimproved sepsis) (*n* = 20).

The comparative analysis was conducted between the first and the second monitoring evaluations for each patient in both groups (Tables 5 and 6). Improved sepsis patients showed significantly different values between both initial and follow-up evaluations regarding nCD64%, hs-CRP, PLT, nCD64 MFI, ANC, Hb, and nCD11b MFI, and this was evident by the high *Z* values and the low *p* values for each of them. While for nonimproved sepsis patients, all measured sepsis markers showed nonsignificant differences (> 0.05) between both evaluations concluding their usefulness in the follow-up of nonimproved sepsis.

### 3.2. Delta Change (dC) Percent Statistical Evaluation

The comparison was conducted between both groups in terms of dC percent (Table 7). The percentage change reflects how big the change is relative to the initial value, and it was calculated for each biomarker by using the following equation: [*X*(final) − *X*(initial)]/*X*(initial)∗100, where *X* stands for the sepsis parameter result.

Each of the following biomarkers could achieve a promising result in the follow-up purpose of sepsis patients: nCD64% (dC *Z* value −5.904), PLT (dC *Z* value −2.899), and hs-CRP (dC *Z* value −2.874). These biomarkers could help in the identification of both clinically improved and continued sepsis patients at the same time. On the other side, dC *Z* values for nCD11b% and nCD11b MFI were −0.567 and −0.397, respectively. These findings highlight the unsatisfactory role of nCD11b in the follow-up and monitoring of neonates with sepsis for both neonates with improved and nonimproved sepsis, simultaneously.

### 3.3. Predictive Performance of Sepsis Biomarkers for 28-Day Mortality

As a secondary objective after analyzing the diagnostic, prognostic, and monitoring performance, the predictive performance of the sepsis markers nCD11b and nCD64 was evaluated (i.e., predicting the patient's mortality based on his preliminary test evaluation result). The valuable predictive capability can greatly assist clinicians in tailoring their appropriate management protocol from the start and allowing them to consider which patients have a potential risk and thus require careful observation. The subjected patients were reclassified according to their outcome into the survivors' group (87) and the nonsurvivors group (23). The predictive validity tests for each parameter were calculated, and a comparison between each of them was conducted (Table 8).

#### 3.3.1. Blood Culture Results

**Figure 3:**
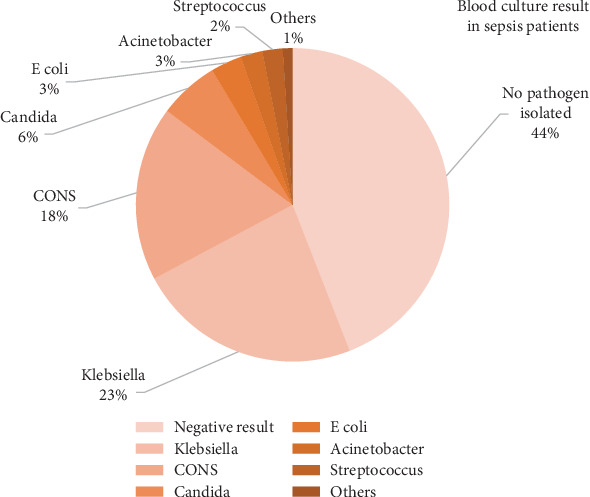
Blood culture results in sepsis patients.

Blood culture was positive in 56% of all sepsis neonates; *Klebsiella pneumoniae* was the most common microorganism isolated (23.1%), followed by coagulase-negative *Staphylococci* (CONS) (18%), and less commonly encountered microorganisms: *Candida* spp. (6.2%), *Escherichia coli* (3.2%), *Acinetobacter* (2.25%), *Streptococcus* spp. (2.1%), and others (1%) (Figure 3).

**Figure 4:**
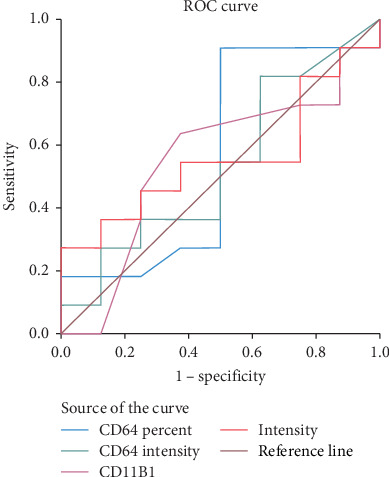
ROC curve analysis for the studied biomarkers between clinically and documented sepsis subgroups.

A comparative statistical analysis was done between documented and clinical sepsis groups.  Table 9 demonstrates the diagnostic performance of the main studied laboratory parameters between both studied groups (Figure 4).

## 4. Discussion

Neonatal sepsis is a major medical problem associated with a high economic burden and multiple medical complications besides the high mortality rates; 21% of enrolled sepsis patients in the current study died from sepsis-related complications. These results are in line with other study results from the same region [22, 23].

The incidence of neonatal septicemia is different from one country to another and from one region to another depending on the presence or absence of sepsis risk factors. Among many documented sepsis risk factors, PROM is one of the proven sepsis predisposing factors through ascending routes of infections [7]. In the current study, PROM was more pronounced among sepsis neonates, as 16% of the sepsis group versus 3% of the control group had PROM, while the duration in the sepsis group was more than 24 h from the time of the mother's delivery; these results are in line with other reported results regarding significant sepsis risk factors [21, 24].

Concerning the blood culture results, almost half the number of the studied sepsis group had positive blood culture results (sensitivity 56%), and several studies reported almost similar results regarding the blood culture diagnostic performance in neonatal sepsis diagnosis [22, 25, 26]. The most common organisms isolated from sepsis neonates in the current study were *Klebsiella* spp. and CONS, and similar culture results were documented by other studies [6, 22, 27].

Regarding the hematological indices as the main parts of the laboratory sepsis profile, there was a significant increase in TLC and ANC in the sepsis patients (LOS group) (*p* = 0.015 and 0.033, respectively) comparable to the controls. While Hb values were significantly lower in sepsis patients (mean = 13.0 gm/dL) than in the controls (mean = 16.0 gm/dL). Thrombocytopenia was significantly expressed in neonates presented by EOS comparable to the controls (*p* value 0.048). It is reported that platelet count is the most affected hematologic parameter in neonatal sepsis, affecting up to 35% of neonates. Although platelet count is not very specific or sensitive, it could help in the diagnosis and follow-up of the course of the disease [28, 29].

Early in the inflammatory response, the occurrence of cytokine-induced activation of leukocytes is evident with a concomitant increase of cell surface molecule expression. During the infections, CD11b and CD64 expressions enhance neutrophil and monocyte migration and trigger various important immune functions. In the present study, CD64%, CD64 MFI, and CD11b MFI showed a higher significant difference when sepsis neonates were compared to the controls.

Concerning nCD64 results, the sepsis group as a whole group and as two separate subgroups (EOS and LOS) had a threefold increase in its expressions when compared to noninfected neonates. Additionally, CD64% had differentiated either EOS or LOS from the control group (*p* values 0.001 and < 0.001, respectively), but it could not differentiate EOS from LOS (*p* value 0.196). CD64% achieved 92.8% sensitivity and 90.8% specificity, respectively; these results come in line with other reported study results regarding the valuable role of CD64 as an early diagnostic neonatal sepsis marker, especially when combined with other acute phase reactants as CRP measurement [8, 30, 31]. Ng et al. [32] reported that CD64 expression was a very sensitive and moderately specific diagnostic tool. On the opposite side, other studies by Layseca-Espinosa et al. [33] reported that CD64 expression had less ideal statistical performance, recording specificity and sensitivity of 25.8% and 96.8%, respectively.

Regarding CD64 MFI in the current study, it showed a higher value in EOS as compared with the controls (*p* = 0.036), but an insignificant difference existed when LOS was compared with EOS (*p* = 0.30) and with the controls (*p* = 0.14). So, it could not differentiate LOS from EOS; in addition, it achieved less ideal (moderate) diagnostic accuracy (72.5% sensitivity and 54.4% specificity). Contrary to our results, Genel et al. [34] documented that CD64 MFI was a sensitive marker to differentiate EOS from the LOS, where it was higher in the earlier group as compared with the LOS group.

Regarding the comparison between clinically and documented sepsis groups, CD64% cannot discriminate between both groups as a nonsignificant difference was illustrated (*p* = 0.398) with low diagnostic performance for it and other studied parameters: CD64 MFI, CD11b%, CD11b MFI, and hs-CRP. This can be attributed to the low diagnostic performance of the blood culture (sensitivity 56%), which is comparable to the blood culture results in other literature [22, 25, 26]. The clinical sepsis patients in the current study already had high levels of sepsis biomarkers and clinically in sepsis according to the international sepsis scores but no organism isolated from the microbiological culture.

Regarding the correlations between the studied cell surface markers and the demographic data of patients in the current study, nCD64 expression was not affected by gender or GA; similarly, studies found that CD64 expression is not affected by newborn maturity [34].

Concerning CD11b expression, CD11b MFI could differentiate between either EOS or LOS from the controls, but its diagnostic performance results were lower than expected, achieving 59.1% and 69.4% for sensitivity and specificity, respectively. While nCD11b% had lower values in both EOS and LOS groups than in the control group, and this comes in agreement with other studies that found that expression of CD11b was lower in neonates with sepsis than in the control group [7, 33, 35], but it did not show a significant difference between the cases and controls groups (*p* > 0.05) and could not differentiate either of EOS or LOS from the control group. This comes in line with Fitrolaki et al. [26] and Markic et al. [36], who reported that no significant difference in CD11b levels between sepsis neonates and healthy controls especially when the latter compared CD11b expression versus other sepsis cell surface markers including nCD64. Turunen et al. [25] mentioned that the role of CD11b in neonatal infections is still debatable, explaining their findings by its widespread sensitivity and specificity between studies that were affected by other conditions such as respiratory distress syndrome.

On the opposite side, Weirich et al. [35] and Nupponen et al. [37] considered CD11b a highly effective marker for diagnosing early-onset neonatal infection. This could be explained by the activation of CD11b expression on neutrophils, which occurs early in the disease process. The delay in recognition of sepsis and, consequently, in obtaining the blood sample might result in missing the peak of CD11b expression. Additionally, the initiation of appropriate antibiotic therapy before measuring CD11b could also lower its results and diagnostic performance. Furthermore, the different studied populations, their different clinical settings, and the existence of different pathological covariates could affect the overall study results.

By comparing the diagnostic validity of nCD11b with nCD64, Fitrolaki et al. and Du et al. [26, 29] reported that nCD64 had better diagnostic performance and more predictive power in neonatal sepsis than CD11b, and these results agreed with our present study results.

Regarding hs-CRP results, it could differentiate EOS and LOS from the control group (both had p <0.001) and was able to differentiate EOS from LOS (p =0.047). This could be explained by the late onset of CRP rising levels: within 6–8 h of infection and peaks at 24 h of the sepsis course. Regarding hs-CRP specificity and positive predictive value, it was 78.15% and 75.7%. These results are supported by Ershad et al.'s [38] results, who reported that CRP may not be elevated in the early stages of infection due to the time taken for its synthesis in the liver and eventual appearance in the blood. Serial measurements of CRP combined with other acute phase reactants and diagnostic sepsis markers will improve its overall diagnostic accuracy [39].

Gilfillan and Bhandari [30] reported that CRP has variable sensitivities and specificities between the studies, with median values of 49%–68% and > 90%, respectively. It rises 12–24 h after infection, levels peak at 36–48 h, then drop more rapidly with antibiotic treatment. It is readily available with a reasonable TAT and high specificity, and levels fall more steeply with effective treatment. On the other side, CRP rises late in the time course of infection, so single measures early in the course of the illness are not reliable or sufficient; besides, its interpretation can be confounded by other physiological and pathophysiological conditions like the mode of delivery.

The current study showed that CD64%, besides hs-CRP measurement, has higher sensitivity, specificity, and positive predictive values. Many studies investigated the diagnostic performance of a combination of sepsis marker measurements. Among these studies, Genel et al. [34] showed that CRP and CD64 had a higher sensitivity (81% and 81%, respectively) and negative predictive values (77% and 75%, respectively) compared to CD11b and CD62, and they concluded that the use of multiple markers would be associated with higher sensitivity and better negative predictive values. Sheneef et al. [40] confirmed that CD64 combined with CRP could improve the sensitivity and specificity of neonatal sepsis diagnosis. Additionally, Song et al. [41] conducted a meta-analysis concerning the diagnostic value of nCD64 combined with CRP for neonatal sepsis, and they reported that the combined application of CD64 and CRP improved the accuracy of neonatal sepsis diagnosis with a sensitivity of 0.95 (95% CI: 0.86–0.98) and specificity of 0.86 (95% CI: 0.74–0.93), respectively.

Concerning the diagnostic efficacy of combined measurement of both CD64 and CD11b, Genel et al. [34] reported that the combination of CD64 with CD11b and CRP further enhances the sensitivity of the expression and the negative predictive value. While this was not the case in our study, the combined measurement of both cell surface biomarkers (CD64 and CD11b) did not achieve a desirable diagnostic performance for sepsis diagnosis (S1).

The monitoring capability of each of the studied sepsis parameters was evaluated in addition to their diagnostic and prognostic performance. Clinically, studied neonates were divided into two groups: improved and nonimproved sepsis. The dC results reflect the unsatisfactory performance of nCD11b comparable to nCD64 in monitoring purposes of sepsis patients.

It seems that there is no single sepsis marker that one can point to as the ideal single marker capable of meeting all our diagnostic and prognostic targets each time. Rather, a combination of more than one sepsis parameter can enhance the diagnostic performance and accuracy of each other. Additionally, the application of rapid blood culture diagnostic techniques, the rational use of antibiotics, and the application of rapid new diagnostic technologies like flow cytometry in sepsis diagnosis, along with the interpretation of all laboratory and radiological results in the context of the patient's clinical conditions, will undoubtedly reduce the high mortality rates and childhood disabilities associated with neonatal sepsis in our region.

Last but not least, there are a few challenges we encountered when implementing the routine use of valuable CD markers in NICUs. Firstly, the TAT of the test during the holidays and nonworking hours, CD64, was only measured during regular business hours, and there was no quick and precise point-of-care (POC) tool that could measure it from a minute blood sample. Neonatal sepsis is an urgent situation that requires test availability around the clock for ongoing monitoring and assessment. Second, the test's price; a CD marker costs 120 L.E. per test, while a CRP test only costs 50 L.E. On the other side, Hassan et al. recently constructed a robust biochip [42]. At the patient's bedside, this biochip may be utilized to measure nCD64 for ongoing monitoring and appropriate assessment. However, CD markers are more expensive than traditional sepsis markers. But the potential savings from avoiding unnecessary organism typing, changing the antibiotics prescribed, detecting false blood culture results, and screening for antibiotics, as well as the reduction in nursing care costs, bed charges, treatment complications, and antibiotic resistance overall, will more than offset the cost of using this target new biomarker.

## 5. Conclusion

CD64%, CD64 MFI, and CD11b MFI are increased in sepsis neonates compared to the healthy controls. nCD64 expression has superior diagnostic performance results compared to nCD11b expression. hs-CRP could differentiate between sepsis and control groups and between either EOS or LOS groups, but with moderate sensitivity and specificity results. Combined nCD64 and hs-CRP measurement will provide an easy, rapid, and accurate early diagnostic modality for sepsis diagnosis, even before the results of the initial blood culture, which can be routinely applicable in NICUs for better patient outcomes. It is statistically evident that nCD11b is less ideal to be used in the routine daily sepsis evaluation compared to nCD64 as a diagnostic, prognostic, or monitoring sepsis marker in NICUs.

### 5.1. Limitations of the Study

Limitations can be addressed. (1) Neonates with congenital anomalies, chromosomal abnormalities, and those who underwent surgical interventions were not excluded from the study. Indeed, this heterogeneity was intended to test the clinical application of sepsis biomarkers in the different heterogeneous groups of patients, which reflect the daily struggles of NICUs. (2) The selection of sepsis neonates, once clinically suspected and before antibiotic therapy had started, was a matter of challenge, especially in a tertiary referral hospital like Ain Shams University Hospital, where many neonates had already received initial antibiotic therapy. (3) The unavailability of flow cytometric analysis after the routine morning hours and on holidays, along with the inability to work with blood samples except for adequate fresh neonatal samples collected within 48 h, all of these points added struggles and challenges to our study. (4) Concerning procalcitonin, it was not included in our comparative study; however, it is well known to be a powerful diagnostic and prognostic biomarker in neonatal septicemia for two main reasons. Firstly, it would be difficult to perform routinely in our hospital because of financial issues and limited resources. Secondly, several studies compared procalcitonin with CD64 for neonatal sepsis diagnosis and monitoring purposes; their results revealed better performance of nCD64 over procalcitonin or even the same. Therefore, it was intended in the current study to compare both new biomarkers, CD64 and CD11b, together to decide which of them is the best.

## Figures and Tables

**Table 1:** Comparative statistics between the three studied groups regarding the demographic and laboratory parameters.

	**Control**	**Early sepsis**	**Late sepsis**	**Test of sig ANOVA (** **F** **)/Kruskal–Wallis test (** **H** **)**	**p** ** value**	**Pairwise comparison** **p** ** value**		
	**Median (IQR)/mean (SD)** ^**a**^	**Median (IQR)/mean (SD)** ^**a**^	**Median (IQR)/mean (SD)** ^**a**^			**Control vs. early onset**	**Control vs. late-onset**	**Early onset vs. late-onset**
GA	37.36 (2.6)	33.33 (3.01)	35.57 (2.54)	*F* = 8.604	**< 0.001** ^**a**^	**< 0.001** ^**a**^	0.060	0.560
WT	2759.0 (680.12)	2239.16 (938.72)	2518.68 (1011.94)	*F* = 1.547	0.222	—	—	—
Age at the study	1 (1–2.5)	7 (1–17.25)	6 (2–12)	*H* = 17.160	**< 0.001** ^**a**^	0.406	**< 0.001** ^**a**^	**0.007** ^**a**^
DOH	1 (0–7)	49 (17.75–78.75)	14 (10–36)	*H* = 23.990	**< 0.001** ^**a**^	**< 0.001** ^**a**^	**< 0.001** ^**a**^	0.769
Hb (g/dL)	16.0 (4.77)	12.08 (3.74)	13.36 (2.94)	*F* = 3.517	**0.053** ^**a**^	**0.0510** ^**a**^	0.085	0.561
TLC (×10^9^/L)	14.3 (10.9–17.2)	13.85 (10.275–31.45)	19.6 (15–23.6)	*H* = 6.890	**0.032** ^**a**^	0.194	**0.011** ^**a**^	0.313
ANC (×10^9^/L)	5.5 (2.85–11.75)	5.9 (3.15–33.1)	12.8 (8.05–18.45)	*H* = 6.706	**0.035** ^**a**^	0.498	**0.010** ^**a**^	0.139
PLT (×10^9^/L)	235 (163–347)	150 (25.5–235)	264 (73–465)	*H* = 1.293	0.524	—	—	—
hs-CRP (mg/L)	0 (0–6)	36 (12–79.25)	12 (6–24)	*H* = 20.979	**< 0.001** ^**a**^	**0.003** ^**a**^	**< 0.001** ^**a**^	0.359
nCD64 (%)	24% (14%–37.5%)	86.8% (80.2%–96.0%)	75% (55.4%–93.1%)	*H* = 25.906	**< 0.001** ^**a**^	**< 0.001** ^**a**^	**< 0.001** ^**a**^	0.861
nCD64 MFI	1.38 (1.285–1.9)	1.94 (1.57–3.25)	1.46 (1.31–2.34)	*H* = 5.825	**0.056**	—	—	—
nCD11b (%)	98% (94.7%–99%)	96.8% (68.2%–98.4%)	96.6 (94.0–98.9) %	*H* = 1.236	0.539	—	—	—
nCD11b MFI	6.47 (5.205–7.34)	3.975 (2.355–6.07)	4.125 (2.9–4.82)	*H* = 17.390	**< 0.001** ^**a**^	**< 0.001** ^**a**^	**< 0.001** ^**a**^	0.910

*Note:* Values are presented as median (IQR) and/or mean (SD). Significant values have been adjusted by the Bonferroni correction for multiple tests.

Abbreviations: ANC, absolute neutrophil count; DOH, duration of hospitalization; GA, gestational age; Hb, hemoglobin; hs-CRP, highly sensitive CRP; nCD11b%, neutrophil CD11b%; nCD11b MFI, nCD11b mean fluorescence intensity; nCD64%, neutrophil CD64%; nCD64 MFI, nCD64 mean fluorescence intensity; *p*, probability value; PLT, platelet; TLC, total leukocytic count; WT, weight.

^a^Mean (SD).

**Table 2:** Diagnostic performance of studied laboratory parameters arranged in terms of their efficacy.

**Marker name**	**Cutoff**	**Sensitivity**	**Specificity**	**PPV**	**NPV**	**Eff.**	**AUC**	**SE**	**95 CI**	
nCD11b%	98.7%	31.8	73.6	42.7	63.1	54.7	0.405	0.084	0.241	0.570
Blood culture	+ve result	50	90	93.8	37.5	60	NA	NA	NA	NA
ANC	5.5	70.6	53.5	65.2	57.8	63.6	0.528	0.052	0.494	0.696
nCD11b MFI	4.64	59.1	69.4	46.4	79.1	66.2	0.144	0.058	0.032	0.257
nCD64 MFI	1.437	72.5	54.4	70.1	56.8	66.3	0.634	0.081	0.476	0.793
hs-CRP	6	69	78.15	75.7	68.6	72.8	0.586	0.051	0.494	0.696
nCD64%	44.15%	92.8	90.8	91.2	92.1	91.8	0.894	0.048	0.800	0.987
nCD64% and CRP	CD64% at 43.7% and CRP at 6 mg/L	93.9	97.2	97.3	88.8	94.46	0.938	0.030	0.876	0.997

*Note:* 95 CI, confidence interval of AUC.

Abbreviations: ANC, absolute neutrophil count (×10^9^/L); hs-CRP, highly sensitive CRP (milligrams per liter); NA, not available; nCD11b%, neutrophil CD11b%; nCD11b MFI, nCD11b mean fluorescence intensity; nCD64%, neutrophil CD64%; nCD64 MFI, nCD64mean fluorescence intensity; SE, standard error.

**Table 3:** Multiregression analysis of the studied laboratory parameters.

**Multiregression analysis:**					
**Dependent variable: Sepsis vs. control:**					
	**Reg. coef.**	**T**	**p**	**F** **-ratio**	**p**
(Constant)	−0.374	−2.479	0.019		
TLC	0.03	2.057	0.049		
hs-CRP	0.001	0.787	0.433		
CD64%	1.258	7.603	< 0.001		
ANC	−0.025	−1.673	0.106		
				**24.001**	**< 0.001**

*Note:* Bold data implies a significant value.

**Table 4:** Comparison between severe sepsis/septic shock patients and patients with nonsevere sepsis.

**Wilcoxon's rank sum test:**						
		**Median**	**25%**	**75%**	**Z**	**p**
GA	Severe sepsis	34	32.75	37		
	Nonsevere sepsis	36	34	38	−2.192	0.028
BW	Severe sepsis	2700	2000	3200		
	Nonsevere sepsis	2580	1900	3212.5	−0.076	0.939
Hb	Severe sepsis	11.15	9.8	12		
	Nonsevere sepsis	13.1	10.675	14.8	−3.507	< 0.0001
TLC	Severe sepsis	10.65	8.15	17.8		
	Nonsevere sepsis	14.6	9.65	19.95	−2.17	0.03
ANC	Severe sepsis	6.5	2.4	12.4		
	Nonsevere sepsis	7	4.4875	12.225	−1.11	0.267
ALC	Severe sepsis	2.7	1.225	4.35		
	Nonsevere sepsis	5	3.4	8	−3.853	< 0.0001
AMC	Severe sepsis	0.9	0.4	2		
	Nonsevere sepsis	1.5	0.8	2.325	−2.517	0.012
PLT	Severe sepsis	90	29	168		
	Nonsevere sepsis	240	158	321.75	−5.252	< 0.0001
hs-CRP	Severe sepsis	2.4	0.975	9.6		
	Nonsevere sepsis	1.2	0.6	3.7	−2.368	0.018
nCD64%	Severe sepsis	94.1	83	97.6		
	Nonsevere sepsis	81.85	60.075	89.375	−4.213	< 0.0001
nCD64 MFI	Severe sepsis	2.15	1.76	3		
	Nonsevere sepsis	1.82	1.44	2.41	−2.958	0.003
nCD11b%	Severe sepsis	98.65	96.475	99.775		
	Nonsevere sepsis	99.15	97.1	99.7	−1.006	0.314
nCD11b MFI	Severe sepsis	3.6	2.79	5.4725		
	Nonsevere sepsis	5.53	4.3025	7.21	−3.322	0.001

**Table 5:** The comparison between the baseline and the follow-up evaluations for the clinically improved groups.

**Wilcoxon's signed rank test:**						
**Among improved:**						
		**Median**	**25%**	**75%**	**Z**	**p**
Hb	First evaluation	12.7	10.4	14.8		
	Second evaluation	11.6	10.2	13	-2.212	**0.027**
TLC	First evaluation	13.5	9	18.2		
	Second evaluation	13	10	15.3	-1.535	0.125
ANC	First evaluation	6.08	4.55	11.15		
	Second evaluation	5.57	3.725	7.075	-2.783	**0.005**
ALC	First evaluation	4.1	2.55	6.95		
	Second evaluation	4.6	3.5	6.05	-0.350	0.726
AMC	First evaluation	1.35	0.7	2.2		
	Second evaluation	0.94	0.5	1.2	-1.859	0.063
PLT	First evaluation	197	106	319		
	Second evaluation	283	200	447	-3.459	**0.001**
hs-CRP	First evaluation	2.3	0.8	4.8		
	Second evaluation	0.6	0.5	2.375	-3.968	**< 0.0001**
nCD64%	First evaluation	87.55	74.5	96.375		
	Second evaluation	35.05	21.5	52.75	-5.511	**< 0.0001**
nCD64MFI	First evaluation	2.37	1.48	3.145		
	Second evaluation	1.45	1.21	1.855	-3.127	**0.002**
nCD11b%	First evaluation	98.7	98.3	99.6		
	Second evaluation	98.9	94.1	99.8	-0.801	0.423
nCD11bMFI	First evaluation	6.17	5.3	7.21		
	Second evaluation	5.39	3.45	5.53	-2.045	**0.041**

*Note:Z*, Wilcoxon's rank sum test; *p*, the probability value. Bold data implies a significant value.

**Table 6:** The comparison between the baseline and follow-up evaluations for the continued sepsis group.

**Continued sepsis group**						
		**Median**	**25%**	**75%**	**Z**	**p**
Hb	First evaluation	12.4	10.85	14.025		
	Second evaluation	11.15	10.3	13.8	-1.349	0.177
TLC	First evaluation	14.7	9.475	18.975		
	Second evaluation	14.3	9.125	23.4	-0.411	0.681
ANC	First evaluation	6.5	3.9	9.1		
	Second evaluation	9.2	4.7	12.5	-1.412	0.158
ALC	First evaluation	4.3	3.115	5.11		
	Second evaluation	2.685	0.8175	3.39	-1.782	0.075
AMC	First evaluation	1.8	1.1	2.6		
	Second evaluation	1	0.45	2.8	-1.014	0.311
PLT	First evaluation	209	120	288		
	Second evaluation	158	88	234	-1.046	0.295
hs-CRP	First evaluation	1.05	0.125	2.2		
	Second evaluation	2.4	0.75	7.95	-1.735	0.083
nCD64%	First evaluation	84.65	57.15	94.075		
	Second evaluation	89	77	94.575	-1.670	0.095
nCD64 MFI	First evaluation	2.115	1.795	3.155		
	Second evaluation	2.69	1.8125	3.4425	-0.806	0.42
nCD11b%	First evaluation	98.8	97.85	99.4		
	Second evaluation	99.3	96.65	99.75	-0.405	0.686
nCD11b MFI	First evaluation	7	4.35	9.17		
	Second evaluation	5.53	4.045	9.045	-0.405	0.686

**Table 7:** Delta change percentage for both the follow-up groups.

**Wilcoxon's rank sum test:**						
		**Median**	**25%**	**75%**	**Z**	**p**
ANC.dC	Improved	−34.821	−53.324	17.16		
	Nonimproved sepsis	20	−28.571	50.602	−1.986	**0.047**
PLT.dC	Improved	44.91	0.295	163.462		
	Nonimproved sepsis	−2.41	−72.24	42.045	−2.899	**0.004**
hs-CRP.dC	Improved	−50	−75	−3.824		
	Nonimproved sepsis	100	−50	300	−2.874	**0.004**
nCD64%dC	Improved	−46.537	−71.605	−34.926		
	Nonimproved sepsis	5.734	−3.855	60.157	−5.904	**0.0001**
nCD64.MFI.dC	Improved	−26.337	−51.262	8.698		
	Nonimproved sepsis	10.264	−28.648	52.582	−2.415	**0.016**
nCD11b%dC	Improved	−0.301	−5.572	1.131		
	Nonimproved sepsis	0.506	−2.766	1.945	−0.567	0.571
nCD11b.MFI.dC	Improved	−22.475	−40.638	8.491		
	Nonimproved sepsis	9.663	−44.489	40.668	−0.397	0.692

*Note:* Bold data implies a significant value.

**Table 8:** The predictive validity results for the studied sepsis markers arranged in ascending order in terms of their sensitivities.

	**Cutoff**	**TN**	**FP**	**TP**	**FN**	**Sp. (%)**	**Sens. (%)**	**NPV (%)**	**PPV (%)**	**Eff. (%)**	**AUC**
nCD11b%	99	94	6	41	41	94.0	50.0	69.6	87.2	74.2	**0.852**
nCD64%	41.6	88	6	126	7	93.6	94.7	92.6	95.5	**94.3**	**0.925**
hs-CRP	0.6	96	6	93	38	94.1	71.0	71.6	93.9	**81.1**	0.575
nCD64 MFI	1.43	50	44	105	25	53.2	80.8	66.7	70.5	69.2	0.574
nCD11b MFI	5.09	64	36	42	40	64.0	51.2	61.5	53.8	58.2	0.522

*Note: *Bold data implies a significant value.

Abbreviations: Eff., efficacy; FN, false negative; FP, false positive; NPV, negative predictive value; PPV, positive predicted value; TN, true negative; TP, true positive.

**Table 9:** Diagnostic accuracy of main studied parameters among documented and clinical sepsis groups.

	**Cutoff**	**AUC**	**TN**	**FP**	**TP**	**FN**	**Sp. (%)**	**Sens. (%)**	**NPV (%)**	**PPV (%)**	**Eff. (%)**
CD64%	74	0.563	5	12	4	1	55.6	92.3	83.3	75	**77.3**
CD64 MFI	1.36	0.534	3	10	6	3	33.3	76.9	50	62.5	**59.1**
CD11 %	97	0.551	4	5	6	7	40	41.7	36.4	45.5	**40.9**
CD11 MFI	4.275	0.557	4	6	6	6	40	50	40	50	**45.5**

*Note: *Bold data implies a significant value.

Abbreviations: Eff., efficacy; FN, false negative; FP, false positive; NPV, negative predictive value; PPV, positive predicted value; TN, true negative; TP, true positive.

## Data Availability

The authors declare that the data underlying the findings of this research are publicly available.
