# Correction to “The Role of Neutrophil CD11b Compared to Neutrophil CD64 as an Early Diagnostic, Monitoring, and Prognostic Sepsis Marker in Neonatal ICUs”

**DOI:** 10.1155/bmri/9793081

**Published:** 2026-07-23

H. E. Hashem, W. O. Ahmed, and S. H. Hassan, “The Role of Neutrophil CD11b Compared to Neutrophil CD64 as an Early Diagnostic, Monitoring, and Prognostic Sepsis Marker in Neonatal ICUs,” *BioMed Research International* 2025 (2025): 7206112, https://doi.org/10.1155/bmri/7206112.

In the above article, the affiliation of author Wafaa O. Ahmed was incorrect. The correct affiliation for this author is as follows:

Pediatric and Neonatology Department, Faculty of Medicine, Ain Shams University Hospitals, Cairo, Egypt

The updated author list and their respective affiliations are as follows:

Heba E. Hashem^1^, Wafaa O. Ahmed^2^, Safeya H. Hassan^1^

^1^Clinical Pathology Department, Ain Shams University Hospitals, Cairo, Egypt

^2^Pediatric and Neonatology Department, Faculty of Medicine, Ain Shams University Hospitals, Cairo, Egypt

We apologize for this error.

